# Integrated metabolome and transcriptome analysis unveils novel pathway involved in the fruit coloration of *Nitraria tangutorum* Bobr.

**DOI:** 10.1186/s12870-023-04076-3

**Published:** 2023-02-01

**Authors:** Huilong Zhang, Aishuang Hu, Haiwen Wu, Jianfeng Zhu, Jingbo Zhang, Tielong Cheng, Sergey Shabala, Huaxin Zhang, Xiuyan Yang

**Affiliations:** 1 Institute of Ecological Protection and Restoration, Chinese Academy of Forestry, Beijing, 10091 China; 2 The Comprehensive Experimental Center, Chinese Academy of Forestry in Yellow River Delta, Dongying, 257000 China; 3 Institute of Coastal Agriculture, Hebei Academy of Agriculture and Forestry Sciences, Tangshan, 063299 China; 4 Experimental Center of Desert Forestry, Chinese Academy of Forestry, Dengkou, 015200 China; 5 Key Laboratory of Forestry Genetics & Biotechnology of Ministry of Education, Co-Innovation Center for Sustainable Forestry in Southern China, Nanjing Forestry University, Nanjing, 210037 China; 6 Tasmanian Institute of Agriculture, University of Tasmania, Hobart, TAS 7001 Australia; 7 International Research Centre for Environmental Membrane Biology, Foshan University, Foshan, 528000 China

**Keywords:** *Nitraria tangutorum*, Anthocyanin, Multi-omics, Regulatory networks, Halophyte

## Abstract

**Background:**

The desert shrub *Nitraria tangutorum* Bobr. is important for its resistance to salt and alkali in Northwest China. It is an ecologically important species in this region and provides edible and medicinal berries. This study showed a mutant of *N. tangutorum* (named Jincan, JC) that has a strong yellow pericarp vs red in a wild type (represented by NT).

**Results:**

In this study, the secondary metabolic and molecular mechanisms responsible for *Nitraria* fruit coloration were investigated using LC–MS-based widely targeted metabolomics and transcriptomics data. As a result of our study, 122 and 104 flavonoid metabolites were differentially expressed throughout the mature and transition stages between JC and NT, respectively. Furthermore, two cyanidin derivatives (cyanidin 3-O-glucoside and cyanidin-3-O-(2''-O-glucosyl) glucoside) and one pelargonidin derivative (pelargonidin-3-O-glucoside) were identified only in the NT phenotype. The functional genes F3H (flavanone 3-hydroxylase), F3'H (flavonoid 3′-hydroxylase) and UFGT (flavonoid 3-O-glucosyltransferase) and the transcription factors MYB, bHLH, NAC and bZIP were significantly downregulated in JC. Meanwhile, the activity of UFGT was extremely low in both periods of JC, with a five-fold higher enzymatic activity of UFGT in RT than in YT. In summary, due to the lack of catalysis of UGFT, yellow fruit of JC could not accumulate sufficient cyanidin and pelargonidin derivatives during fruit ripening.

**Conclusion:**

Taken together, our data provide insights into the mechanism for the regulation of anthocyanin synthesis and *N. tangutorum* fruit coloration and provide a theoretical basis to develop new strategies for developing bioactive compounds from *N. tangutorum* fruits.

**Supplementary Information:**

The online version contains supplementary material available at 10.1186/s12870-023-04076-3.

## Background

*Nitraria tangutorum* Bobr. is a highly valuable species in China and an important constructive shrub species distributed in saline and saline-alkali lands [1–3]. As a source of edible berries and medicinal materials, the fruit of *N. tangutorum* has high potential economic value [4–8]. *N. tangutorum* fruits are often used in non-traditional medicine in Gansu Province, Inner Mongolia and Xinjiang Uygur Autonomous Region. Usually, *N. tangutorum* has red or purplish red ripe fruit. Our group successfully selected a mutant of *N. tangutorum* and named it ‘Jincan’. Compared with the original type, ‘Jincan’ has bright yellow fruit and late flowering. Among these traits, yellow fruit is the most representative character. Fruit colour is linked to genetic traits with ecological value and important functions in attracting seed dispersers [9]. Meanwhile, it provides information on maturity, nutrition, and potential health benefits [10]. Accordingly, identifying the effective metabolic components of ‘Jincan’ will be conducive to the further development and utilization of *Nitraria* fruit.

The colour of fruit is an important characteristic that has a significant impact on its development, utilization, and commercial value. Fruit can show colourful colours because there are many metabolites associated with fruit color, such as pigments, aromatic compounds and tannins [11–14]. In most plant organs, color presentation is primarily determined by the type and content of anthocyanins that belong to flavonoids. For example, pelargonidin, cyanidin and delphinidin, which are the main anthocyanin pigments, are usually found in deep-colored organs in the form of glycosylation [15]. Currently, the anthocyanin biosynthetic pathway is well understood [15–17]. In the first step, phenylalanine is catalyzed by PAL (phenylalanine ammonia lyase), C4H (cinnamate-4-hydroxylase) and 4CL (4-coumarate CoA ligase) to produce *P*-coumaroyl-CoA, and this process is present in many secondary metabolic pathways. Next, dihydrokaempferol is synthesized from *P*-coumaroyl-CoA as a substrate catalyzed by CHS (chalcone synthase), CHI (chalcone isomerase), and F3H (flavanone 3-hydroxylase). In the third step, dihydrokaempferol is catalyzed by DFR (dihydroflavonol 4-reductase) for the synthesis of leucoanthocyanidins, which are then catalyzed by ANS (anthocyanin synthase) to produce the corresponding colored anthocyanins. Finally, the modification of anthocyanins is completed by UFGT (flavonoid 3-O-glucosyltransferase) to form stable anthocyanins, and this is the key step in anthocyanin biosynthesis. However, there is no one-size-fits-all rule that applies to plants because there are always commonalities and characteristics among different plants. Because of the great differences in metabolic species and genes regulating pigments in different plants, it is necessary to analyze different plants individually.

With the development of sequencing technologies, integrated analysis based on multi-omics has been widely proven to be a useful method to elucidate the regulatory mechanisms of plant biological properties and environmental responses [18–21], such as the salt stress response [22] and the reproduction strategy adapted to the environment [23]. In addition, integrated metabolomics and transcriptome analysis has been widely applied to explore the signaling pathways and mechanisms that regulate pigment enrichment in plant pulp or pericarp [13, 14]. Therefore, combined transcriptome and metabolome analysis represents a promising approach to study the formation of fruit color differences in two *N. tanguturum* phenotypes.

In the present study, to clarify the different regulatory networks among two *N. tanguturum* phenotypes with different fruit colors and the genes that responded to fruit color regulation, four samples from two phenotypes (wild type with red fruit NT and mutant with yellow fruit JC) and two different periods were analyzed. They were red fruits in a transition period (RT) and red fruits in a mature period (RM) of NT, yellow fruits in a transition period (YT) and yellow fruits in a mature period (YM) of JC, respectively. Metabolome and transcriptome studies were performed using an LC‒MS-based widely targeted metabolomics method and Illumina HiSeq platform, followed by an integrated metabolome and transcriptome correlative analysis.

## Results

### Morphology analysis of the fruit color transitions

**Fig. 1:**
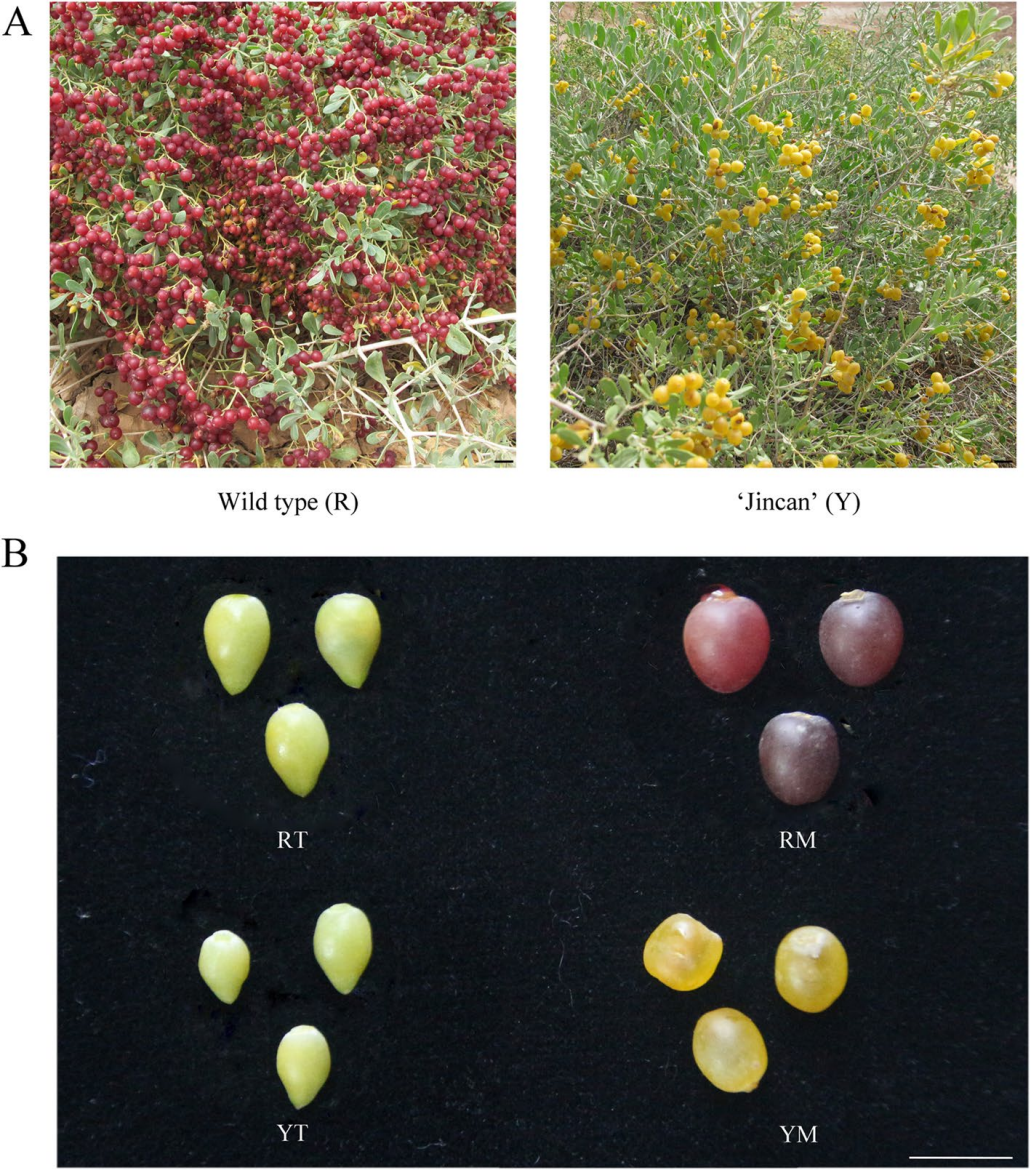
Morphological observation of two phenotypes from *N. tangutorum* Bobr. **A** Yellow fruit phenotype ‘Jincan’ and red fruit phenotype wild type of *N. tangutorum* in a natural growing environment. **B** Phenotype of fruit in YT, YM, RT and RM. Scale bar = 1 cm

NT and JC are two near-isogenic *N. tangutorum* phenotypes. In general, the pericarp of most *N. tangutorum* fruits is red or crimson, and *N. tangutorum* with yellow fruits such as ‘Jincan’ (JC) has never been reported. *N. tangutorum* (NT), with red fruits, is one of the most widely distributed types and is mostly studied (Fig. 1A). During different ripening stages of fruits in *N. tangutorum*, the young fruits of NT and JC were green, and then both of them turned pale yellow. At maturity, the fruit color of NT became red or crimson, while that of JC became bright yellow. RM vs YM, RM vs YT and RM vs RT were three groups of four fruit sample types (RT, RM, YT and YM, Fig. 1B) with different colors, but there was no significant difference between RT and YT.

### Metabolome data quality analysis

**Fig. 2:**
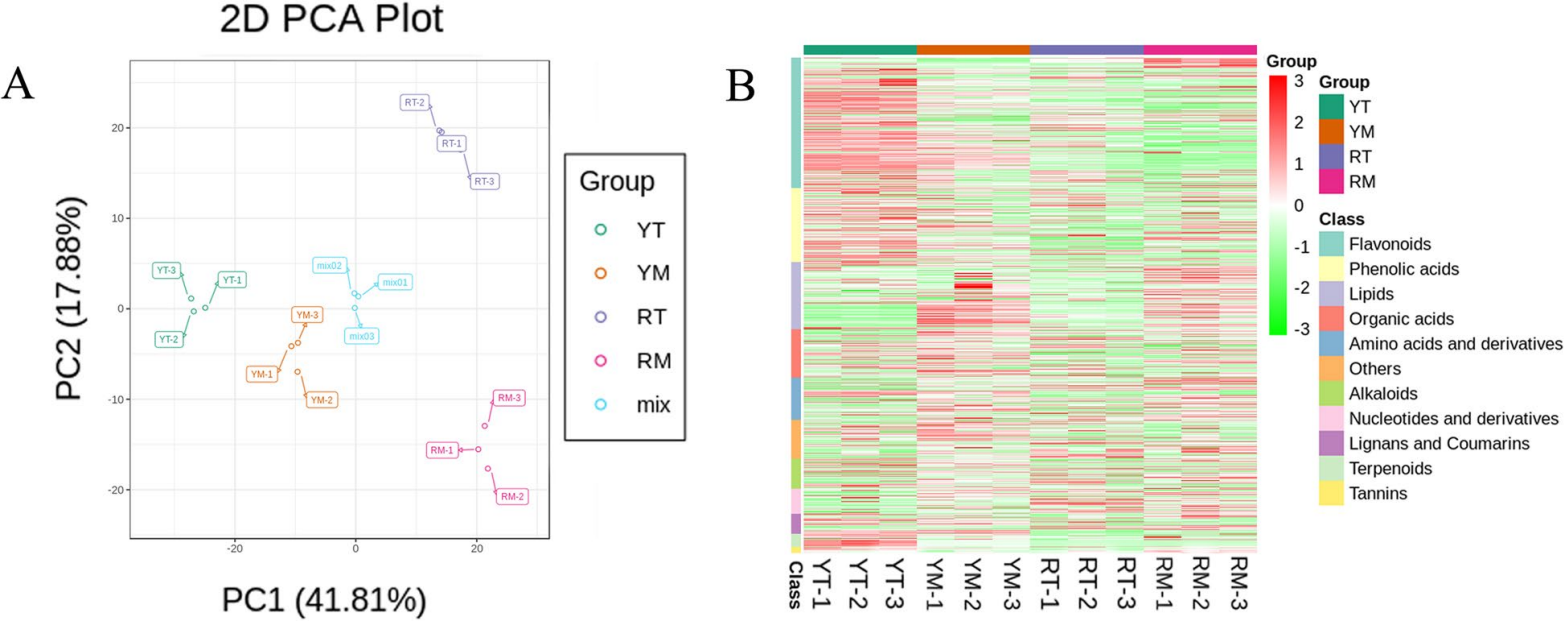
Preliminary analysis of metabolome data. **A** PCA score map. **B** Heatmap of metabolites in all samples

Principal component analysis (PCA) of fruit samples is helpful to characterize the overall variation in metabolism among the samples and the degree of variation among fruit samples and within a group. The PCA diagram showed that there was a significant separation trend in the four different groups presented (Fig. 2A). The two principal components PC1 and PC2 of YT vs RT were 68.05 and 11.05%, and 63.2 and 12.71% between YM and RM. (Supplementary Fig. 1B). The PC1 values were positively correlated with the degree of metabolite variation among different groups. According to the PCA results, significant differences were shown among the four samples. In addition, the most abundant metabolites in all samples were flavonoids, followed by phenolic acids, lipids, organic acids, amino acids and derivatives, others, alkaloids, nucleotides and derivatives, lignans and coumarins, terpenoids, and tannins (Fig. 2B).

### Comparison of metabolites between four fruit samples

**Fig. 3:**
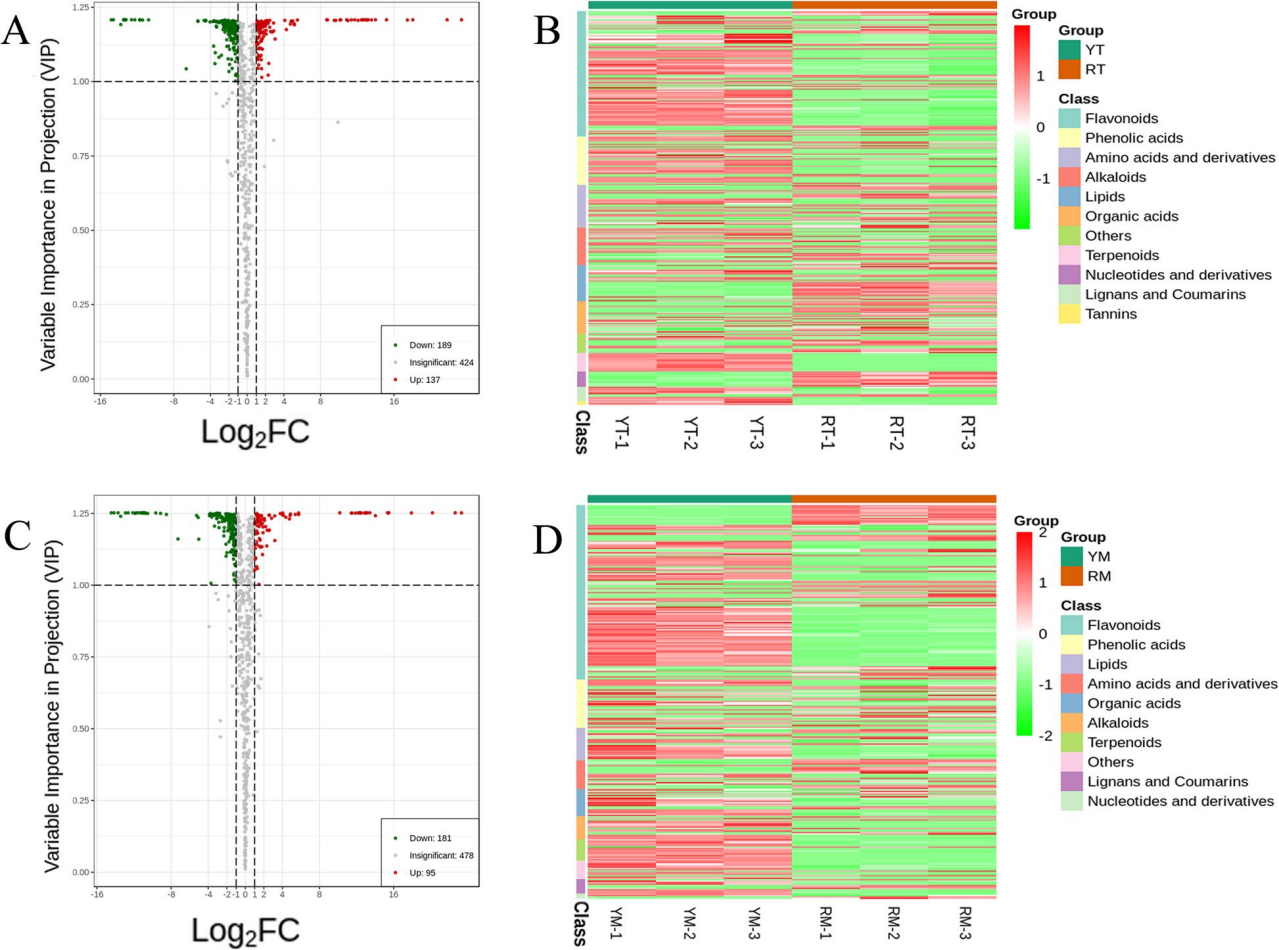
Expression analysis and clustering of metabolites. **A** and **C** Volcano plot of differential metabolites in YT vs RT (**A**) and YM vs RM (**C**); (**B** and **D**) Clustering heatmap of metabolites in YT vs RT (**B**) and YM vs RM (**D**)

In total, 755 metabolites were screened from the four types of fruit samples via UPLC‒MS/MS technology (Supplemental Fig. 2). The raw data collected for all metabolic species detected in this study are shown in Supplemental Table 1. A total of 21 metabolites existed only in the NT phenotype (RT and RM) (Supplemental Table 1), and 14 metabolites were specifically detected in the JC phenotype (YT and YM) (Supplemental Table 1); meanwhile, 683 metabolites were found in both phenotypes. The most abundant metabolites between YM and RM were flavonoids, followed by phenolic acids, lipids, amino acids and derivatives, organic acids, others, alkaloids, nucleotides and derivatives, lignans and coumarins, terpenoids, and tannins (Fig. 3D). In YT vs RT, the highest content of metabolites was flavonoids, followed by phenolic acids, amino acids and derivatives, alkaloids, lipids, organic acids, others, terpenoids, nucleotides and derivatives, lignans and coumarins and tannins (Fig. 3B). This indicated that the difference between JC and NT could be due to the accumulation of flavonoids. Therefore, the following analysis focused on the metabolic pathway of flavonoids.

### Differential metabolites and KEGG annotation

The differentially accumulated metabolites (DAMs) with significant differences were screened from the two groups (YM vs RM and YT vs RT) and presented in a volcano plot (Fig. 3A, C). In YM vs RM, there were 276 DAMs, of which 181 DAMs were downregulated (i.e., 181 DAMs were lower in RM than in YM) and 95 DAMs were upregulated (Fig. 3C). A total of 189 DAMs were downregulated and 137 DAMs were upregulated in YT vs RT (Fig. 3A).

The difference of flavonoids accumulation (DAFs) from DAMs between the two groups was further analyzed. In YM vs RM, 80 DAFs were downregulated and 42 DAFs were upregulated; in YT vs RT, 76 DAFs were downregulated and 28 DAFs were upregulated (Supplemental Table 2). The composition of the DAF with significant differences in each comparison group was examined. Among the upregulated flavonoids, three anthocyanins, cyanidin-3-O-(2''-O-glucosyl) glucoside, cyanidin-3-O-glucoside (kuromanin) and pelargonidin-3-O-glucoside, were detected in the RM and RT samples (Supplemental Table 2). These three metabolites might be the key factors determining the different colors between the two *N. tangutorum* phenotypes.

### Transcriptome sequencing and annotation

Four different types of samples, RT, RM, YT and YM, were collected and analyzed by RNA-Seq. After filtering low-quality reads, this study achieved 600.47 million high-quality reads, with a Q30 > 93.59%. The results of base composition analysis showed that the content of guanine-cytosine (GC) in each sample ranged from 42.72 to 44.99%, and the number of clean bases obtained in each sample was 6.51‒10.04 Gb (Supplemental Table 3). The unigene for subsequent analysis was the longest cluster sequence obtained by Corset hierarchical clustering of transcripts. In total, 307,259 unigenes were obtained with a transcript N50 = 1210 bp, which indicated that the transcriptome was high quality (Supplemental Table 3). These results showed that the present data were reliable and could be used for further analysis.

Functional annotation of all assembled genes was annotated according to BLASTX (E-value < 10^–5^) and based on the public databases GO, Swiss-Prot, NR, COG/KOG, Trembl and KEGG. More than 185,801 unigenes (60.47%) were successfully matched in a minimum of one database (Supplemental Table 4; Supplementary Fig. 3). Of these, 178,161 (57.98%) were tagged in the NR database, and 146,751 (47.76%) were matched to at least one GO term. Unigenes were divided into 25 functional clusters according to the KOG database (Supplemental table 5; Supplementary Fig. 4). In total, 130,038 unigenes were assigned to no less than one KEGG ontology ID and mapped to 142 pathways (Supplemental table 6).

Genes with significant differences in expression were screened for further analysis of the DEGs among the four samples. In the comparison of YT vs RT and YM vs RM, 41,431 (upregulated 20,129, downregulated 21,302) and 23,482 (upregulated 11,772, downregulated 11,710) significantly differentially expressed genes were obtained (Supplementary Table 7; Supplementary Fig. 5). This indicated that there were significant differences between different fruit samples in different colors (YT vs RT and YM vs RM) at the molecular level.

To delve into the possible functions of DEGs, all of them were annotated via the KEGG database, and the significantly enriched KEGG items in DEGs were identified (Supplementary Fig. 6). The KEGG enrichment data of DEGs were analyzed in two comparison groups, YM vs RM and YT vs RT. In total, 28,482 genes were successfully annotated to 139 KEGG metabolic pathways in YM vs RM, and 41,431 genes were successfully annotated to 139 KEGG metabolic pathways in YT vs RT (Supplementary Table 8). The enrichment results showed that the DEGs of the two comparison groups were mainly concentrated in the metabolic process. Moreover, the biosynthesis of phenylpropanoids (287 DEGs), flavonoids (137 DEGs) and anthocyanins (26 DEGs) were significantly enhanced in YT vs RT (Supplemental Fig. 5A; Supplementary Table 8). In the comparison of YM vs RM, phenylpropanoid (216 DEGs), flavonoid (115 DEGs) and anthocyanin (19 DEGs) were significantly changed (Supplementary Fig. 5B; Supplementary Table 8). According to DEG analysis, the difference in fruit color between the two *N. tangutorum* phenotypes may be caused by variation in the expression of genes involved in anthocyanin production.

### Weighted gene correlation network analysis (WGCNA)

**Fig. 4:**
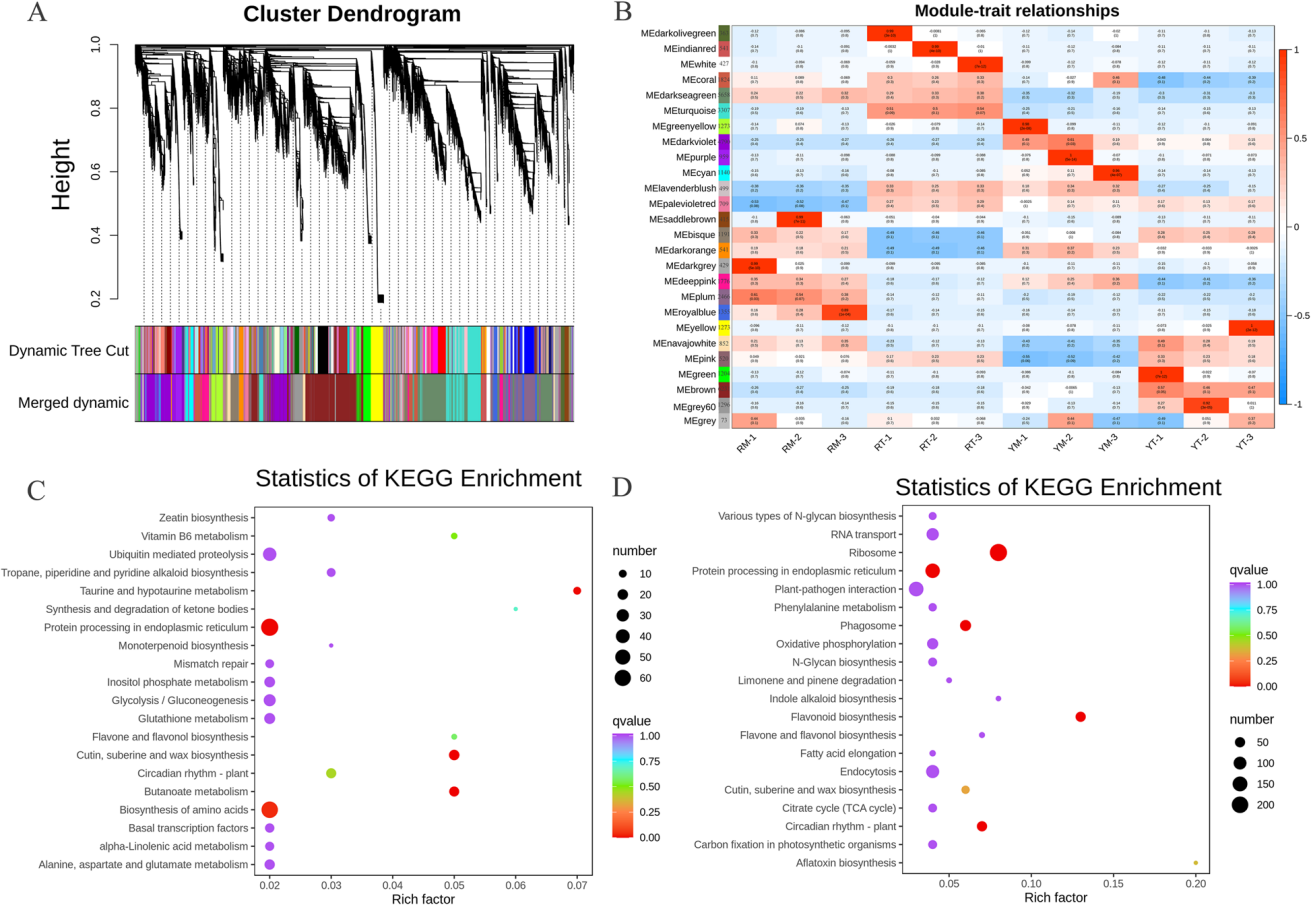
Correlations of structural genes with taste attributes based on WGCNA. **A** Clustering dendrogram of the average network adjacency for the identification of structural gene co-expression modules. **B** Module-trait relationships between fruit color and genes. **C** KEGG enrichment of the plum module. **D** KEGG enrichment of the dark sea-green module

WGCNA was performed to determine hub genes of transcriptional regulation networks highly associated with the fruit color of two *N. tangutorum* phenotypes. The soft-thresholding powers were obtained from 1 to 30 via scale-free topology criteria, and a power of 17 was set to identify modules (Fig. 4A). A total of 26 modules, each with 73–6,321 genes labeled by different colors, were identified (Fig. 4B). Two modules (plum and royal blue) were only positively correlated with RM (Fig. 4B), and the dark sea-green module was positively correlated with the mature and transition stages of red fruit color. These results suggested that genes in the plum, royal blue and dark sea-green modules may have a stronger correlation in different fruit colors than other genes.

KEGG enrichment results showed that pathways associated with the biosynthesis of diverse secondary metabolites, including flavonoids, flavones, anthocyanin, and flavonols, were involved in the plum and dark sea-green modules (Fig. 4C and D). In the plum module, there were 16 genes related to “phenylpropanoid biosynthesis”, 8 genes related to “flavonoid biosynthesis”, and 3 genes related to “anthocyanin biosynthesis”. In the dark sea-green module, there were 50 genes related to “phenylpropanoid biosynthesis”, 35 genes participating in “flavonoid biosynthesis” and 2 genes belonging to “anthocyanin biosynthesis”. The data presented above further indicated that the DEGs in these three modules most likely participated in the formation of fruit color differences between the two *N. tangutorum* phenotypes.

### Structural genes involved in anthocyanin biosynthesis

**Fig. 5:**
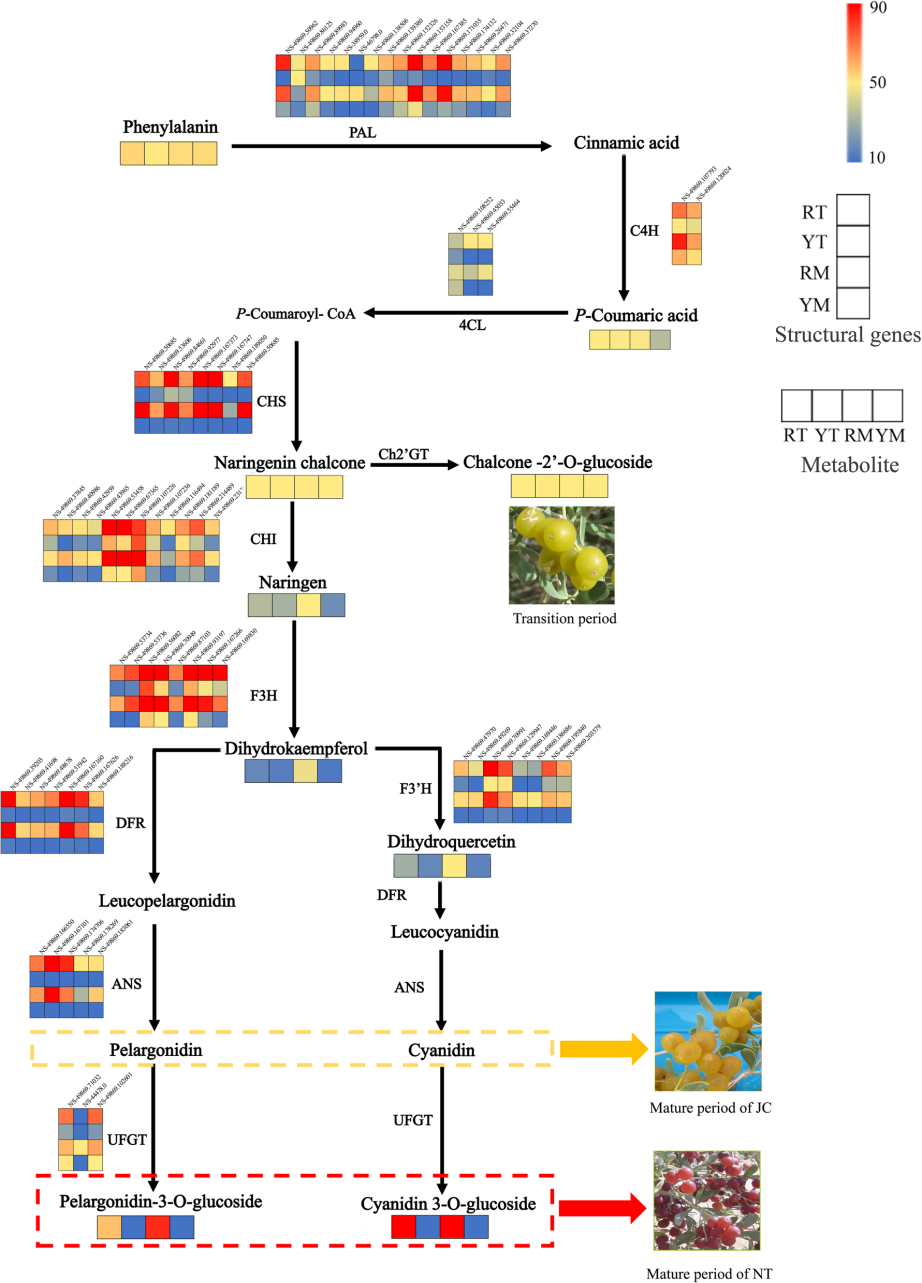
Anthocyanin biosynthesis pathway in the yellow and red fruits of N. tangutorum. The heatmaps indicate the expression/content of respective structural genes/metabolites in two *N. tangutorum* phenotypes. The percentile value of FPKM/relative content values of structural genes/metabolites ranging from low to high is represented by blue to red. The key enzymes in the anthocyanin synthesis pathway [24–26] are shown below: PAL, phenylalanine ammonia lyase; C4H, cinnamate 4-hydroxylase; 4CL, 4-coumarate: CoA ligase; CHS, chalcone synthase; Ch2’GT, chalcononaringenin 2'-O-glucosyltransferase; CHI, chalcone isomerase; F3H, flavanone 3-hydroxylase; F3′H, flavonoid 3′-hydroxylase; DFR, dihydroflavonol 4-reductase; ANS, anthocyanidin synthase; and UFGT, flavonoid 3-O-glucosyltransferase

Based on previous studies on the metabolic pathways of anthocyanins, a pathway schematic chart was drawn that contained the heatmap of each critical gene expression and metabolite content in anthocyanin biosynthesis according to WGCNA of two *N. tangutorum* phenotypes (Fig. 5). Critical genes such as *PAL*, *C4H*, *4CL*, *CHS*, and *CHI* were involved in the initial enzymatic processes of the anthocyanin metabolic pathway (Fig. 5, Supplementary Table 9).

Eight *F3H* genes were identified based on the transcriptome data that were abundantly enriched in RT or RM, and four were almost not expressed in YT and YM (*NS-49869.53734*, *NS-49869.53736*, *NS-49869.87103*, and *NS-49869.169830*) (Fig. 5, Supplementary Table 9). Furthermore, eight upregulated *F3′H* genes were identified in RM and RT (Fig. 5, Supplementary Table 9). In addition, all of the identified *F3′H* genes were almost unexpressed in YM, and four of them (*NS-49869.47970*, *NS-49869.49269*, *NS-49869.169446*, and *NS-49869.186686*) were barely expressed in YT. Therefore, the differential expression of *F3′H* may be an important step in determining which kinds of anthocyanins will be synthesized. Here, seven *DFR* genes upregulated in RM and RT were obtained in the present study. Moreover, all of the identified *DFR* genes were almost unexpressed in YM and YT (Fig. 5, Supplementary Table 9). In this study, five *ANS* genes (*NS-49869.166550*, *NS-49869.167101*, *NS-49869.174706*, *NS-49869.178269* and *NS-49869.183061*) were identified as upregulated genes in RM and RT (Fig. 5, Supplementary Table 9). In wild type*,* three *UFGT* genes were upregulated in RM and RT (*NS-49869.71032*, *NS-44478.0* and *NS-49869.102601*), and one *UFGT*/*3GT* (*NS-44478.0*) gene was only expressed in RM (Fig. 5, Supplementary Table 9).

### Identification of TFs related to anthocyanin biosynthesis

A total of 1,075 TFs with differential expression were found in YT vs RT and 737 in YM vs RM (Supplementary Table 10). In this study, 105 MYB, 39 bHLH and 2 WD40 were identified in YM and RM (supplementary Fig. 5B), and 122 MYB, 68 bHLH and 1 WD40 were detected in YT and RT (Supplementary Fig. 5C). Moreover, nine MYB and five bHLH genes were expressed only in RT, while 15 MYB genes were expressed only in RM, and their absence may be an important factor in the inability of JC to change from yellow to red. In addition, 68 ERF, 58 NAC, 57 WRKY, and 45 bZIP in YT vs RT and 62 ERF, 50 NAC, 41 WRKY, and 25 bZIP in YM vs RM were also detected from DEGs in the previous period.

### Verification of gene expression through qRT‒PCR and UFGT activity analysis

**Fig. 6:**
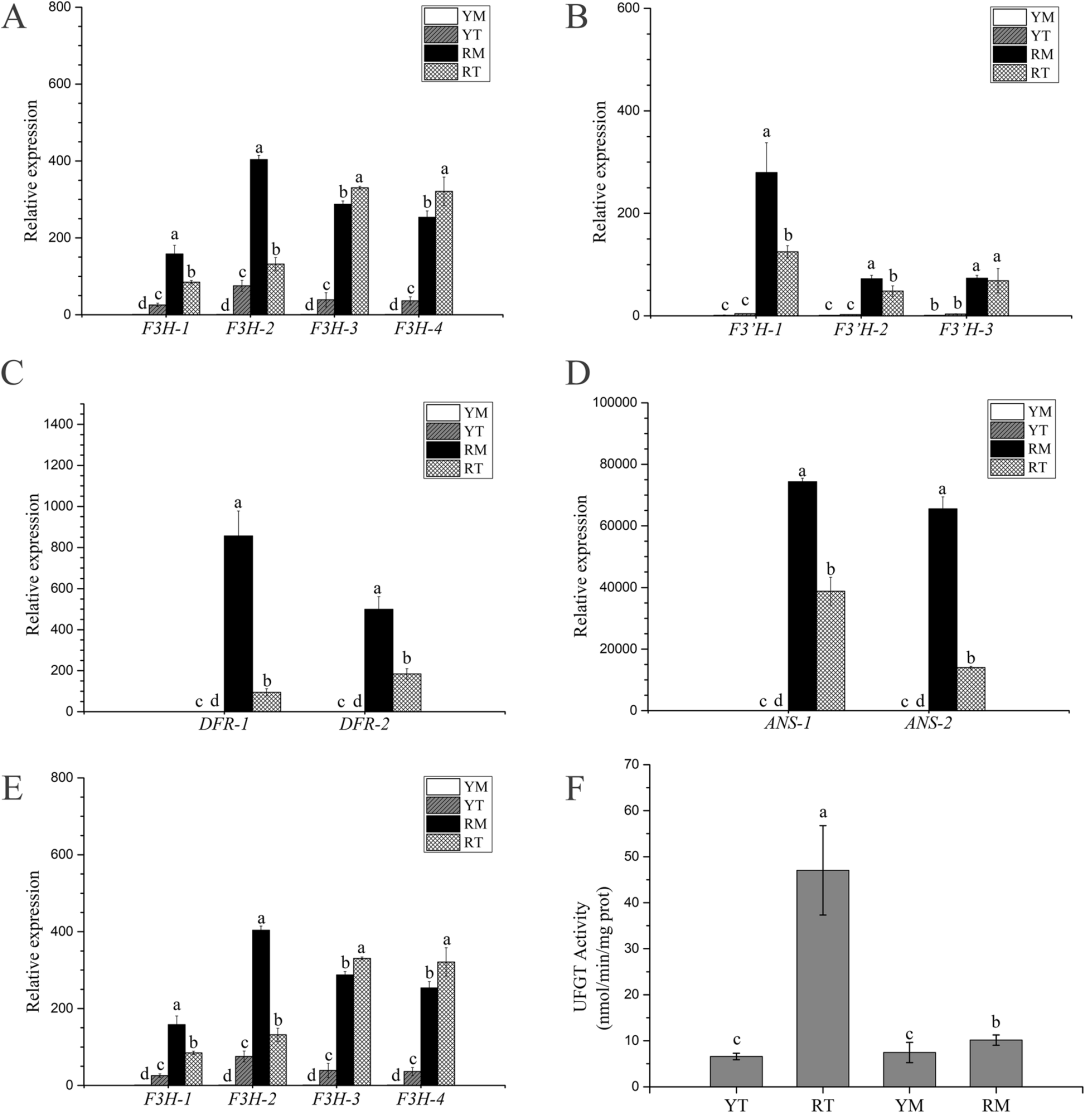
Analysis of critical genes expression and enzyme activity analysis involved in the anthocyanin biosynthesis pathway. **A**-**E** Analysis of the expression pattern of critical genes involved in anthocyanin biosynthesis among four different samples and (**F**) UFGT activity analysis in different fruit samples. Each column shows the average value of three replicated experiments, and bars indicate the standard error of the value. Different letters denote significant differences at *P* < 0.05

To validate the transcriptome data, thirteen encoding genes belonging to five key enzymes with critical roles in anthocyanin biosynthesis were selected for qRT‒PCR verification. The results indicated that all the selected genes were expressed at low levels in JC, which was in accordance with the results of transcriptome data (Fig. 6A-E). In addition, the transcriptome data accurately reflected differences in anthocyanin biosynthesis gene expression between two phenotypes of *N. tangutorum*.

The activity of UFGT was analyzed and the results showed that the activity in NT was notably increased compared with that of JC. There was no significant difference of UFGT activity between YT and YM, but it was appreciably higher in RT than that in RM (Fig. 6F). This suggested that the high activity of UFGT during fruit ripening was necessary to synthesize enough stable anthocyanins, which promoted the transformation of NT fruit from yellow to red.

## Discussion

### Metabolites of the anthocyanin biosynthesis pathway affected the two phenotypes of fruit color

*N. tangutorum* Bobr. is a salt-diluting halophyte widely distributed in north-western China with strong environmental adaptability [1–3]. The yellow fruit phenotype named ‘Jincan’ was obviously different from the wild type in fruit color, which was increasingly regarded as an important factor affecting the external and internal qualities of fruits. In this study, widely targeted metabolomics analysis of fruits was performed to analyze the metabolites involved in fruit coloration. Based on the secondary classification, all 597 identified metabolites were classified into 21 substances (Fig. 3B and D). Among the identified metabolites, there were significant differences between ‘Jincan’ and NT fruits in the contents of flavonoids, particularly in the anthocyanin subclasses (Fig. 5). Flavonoids are generally considered to be key molecules in the formation of plant pigmentation [13, 16, 27]. The present results suggested that the content divergence of flavonoids and the difference in anthocyanins were the most obvious. These differences were the primary reason for yellow or red pigmentation in the two differential color fruits of *N. tangutorum*.

In this work, several subclasses of flavonoids were obtained, including flavanone, flavone, flavonol and anthocyanins. These flavonoids accumulated differently in *N. tangutorum* fruits with different ripening periods and colors (Supplemental table 2). Anthocyanins are essential flavonoid colorants that determine the coloring of plant fruits and protect plants against environmental stress. Meanwhile, these compounds can also promote human health [28]. Previous studies highlight the role of anthocyanins in peel coloration in cucumber and logan [11, 13]. In our study, the results indicated that the glucoside of cyanidin-3-O-(2''-O-glucosyl), cyanidin-3-O and pelargonidin-3-O was found only from the red-colored fruits of *N. tangutorum* based on the metabolome data (Fig. 5, Supplemental table 2). These anthocyanins were candidates for critical metabolites responsible for the pigmentation of *N. tangutorum* fruit. Thus, accumulation of cyanidin and pelargonidin derivatives in the fruit skin conferred the dark red color of NT fruit, which was consistent with previous reports [29]. Therefore, the absence of these three key metabolites may be the main factor that inhibited the JC fruit from changing from yellow to red.

### Structural genes involved in anthocyanin biosynthesis modulated fruit color in *N. tangutorum*

Some structural genes have been shown to participate in the flavonoid/anthocyanin biosynthetic pathway, such as CHS, CHI and F3H, which act as enzymes involved in upstream biosynthesis. *F3′H*, *F3′5′H*, *DFR*, *ANS*, and *UFGT* are usually categorized as anthocyanin-specific biosynthesis genes [16, 17]. The transcript expression of *F3H*, *F3′H*, and *UFGT* were significantly lower in JC fruits than that in NT fruits, even some of these coding genes were almost not expressed in JC fruits (Fig. 5). Additionally, *CHS*, *CHI*, *DFR*, and *ANS* transcription levels in NT differed from those in JC (Fig. 5). The increased expression of *F3H* and *F3′H* leaded to dihydroquercetin accumulated more readily, which was one of the vital precursors eventually to synthesize the stable cyanidin 3-O-glucoside catalyzed by the dual enzymatic DFR and UFGT in the red fruits of wild type. In contrast, the low transcription levels of *F3H* and *F3′H* in the fruit of JC reduced the synthesis of precursors of delphinidin pelargonidin 3-O-glucoside and cyanidin 3-O-glucoside (Fig. 5). This was similar to the results of anthocyanin biosynthesis in torenia (*Torenia fournieri* Lind.) and *Taxus chinensis* [30, 31]. The *UFGT* gene plays a vital role in the synthesis and the final step of the accumulation of anthocyanins, and it is gradually activated along with the fruit coloring period [11, 32]. Unglycosylated anthocyanins are extremely unstable and easily degraded. UFGT, as a key enzyme, can influence the glycosylation process to promote the stability of anthocyanins [15, 32]. The present results showed that the high enrichment of early metabolites in the flavonoid/anthocyanin biosynthesis pathway was triggered by the high expression of related structural genes during fruit discoloration (Figs. 5 and 6). As fruit ripening progressed, the *UFGT* gene converted the metabolites accumulated in the early stage into stable anthocyanins (cyanidin-3-O-glucoside and pelargonidin-3-O-glucoside) involved in red fruit *N. tangutorum* reddening. However, the expression and enzyme activity of UFGT in JC was very low (Figs. 5 and 6), and the two key stable metabolites mentioned above were not found in YT or YM of JC. UFGT has been validated to regulate the synthesis of anthocyanins during the reddening process, such as jujube fruit (*Ziziphus jujuba* Mill.) [14] and *Cerasus humilis* (Bge.) Sok*.* [33]. Therefore, these results proposed that the difference between red fruit and yellow fruit in *N. tangutorum* was mainly due to the process catalyzed by UFGT, in another word, stable anthocyanin glycosides cannot be formed in JC so that the yellow fruit of JC fails to show red coloration. However, more conclusive molecular and genetic data are required to validate the proposed model of transcriptome changes in metabolic profile regulation. The further molecular genetic research on the difference in fruit color between JC and NT will be conducted in our future work.

### Transcription factors regulate genes involved in anthocyanin biosynthesis

Transcription factors play essential roles in a multitude of cellular and developmental processes, such as seedling development, fruit ripening, signal transduction, and various biotic and abiotic stress responses. For example, the MYB-bHLH-WD40 complex (MBW) specifically modulates anthocyanin-specific biosynthetic genes [34–37]. This study identified 105 MYB, 39 bHLH, and 2 WD40 in YM vs RM and 122 MYB, 68 bHLH, and 1 WD40 in YT vs RT according to their expression levels. Based on our research, 68 ERF, 58 NAC, 57 WRKY, and 45 bZIP in YT and RT and 62 ERF, 50 NAC, 41 WRKY, and 25 bZIP were also identified in YM and RM. These TFs were confirmed to regulate the biosynthesis of anthocyanin directly or indirectly via interactions with the MBW complex or anthocyanin-specific structural genes in model plants and other fruits [16, 37–39]. Anthocyanin biosynthesis may be regulated by these differentially expressed transcription factors.

## Conclusions

In summary, the regulatory network of fruit coloration between two *N. tangutorum* phenotypes was explored by using metabolomics and transcriptomics methods. The difference in anthocyanin content, especially the absence of anthocyanin-3-O-glucoside and geranium-3-O-glucoside at the mature stages, was the major force hindering JC fruits from changing from yellow to red. Moreover, multiple regulatory patterns of structural genes and TFs participating in the biosynthesis of anthocyanins were identified. Manipulating UFGT and its regulatory factors (transcription factors) may be the crucial factor leading to the loss of stable anthocyanins in the pericarp and flesh of JC. Color is one of the vital representations of the sensory characteristics of fruits and is becoming increasingly important as the basis for selecting and breeding different fruits and the varieties of these fruits based on expectations of maturity, taste, nutrition, and potential health benefits. The results of this study are helpful to clarify the molecular basis and regulatory mechanism of anthocyanin biosynthesis in ‘Jincan’ and provide a theoretical basis to develop new strategies for developing bioactive compounds from *N. tangutorum* fruits.

## Materials and methods

### Plant materials

Fruits of *N. tangutorum* Bobr. were obtained from saline-alkaline land in Zhangye Danxia National Geological Park of Gansu Province, China. The phenotype ‘Jincan’ (JC) with yellow fruit was compared with wild-type *N. tangutorum* (NT) with red fruit. JC and NT trees (three plants each type) were grown under normal field conditions and the same management. Two developmental stages of fruits, i.e., RT and RM of NT and YT and YM of JC were sampled for further analysis. All fruit samples were stored at -80 °C for RNA-seq and widely targeted metabolomic analysis.

‘Jincan’ was identified by Xiuyan Yang, Institute of Ecological Protection and Restoration, Chinese Academy of Forestry, and a voucher specimen of ‘Jincan’ has been deposited in our laboratory. Jingbo Zhang, Experimental Center of Desert Forestry, Chinese Academy of Forestry, was responsible for wild-type *N. tangutorum* identification, and a voucher specimen of wild-type *N. tangutorum* (number:00013399) has been deposited in the China National Specimen Information Infrastructure, Institute of Botany, The Chinese Academy of Sciences. All materials were collected in accordance with relevant institutional, national, and international guidelines and legislation.

### Widely targeted metabolomic analysis

Widely targeted metabolomic analysis was performed using UPLC (Shim-pack UFLC SHIMADZU Nexera X2) and MS (Applied Biosystems 4500 QTRAP), and the specific details were described in a study by Xia et al. [12]. Unsupervised Principal Component Analysis was performed to assess and validate the content and accuracy of metabolites from all samples. Significantly regulated metabolites among groups were calculated according to the following standards: VIP >  = 1 and | Log_2_FC |> = 1. Total identified metabolites were annotated through the KEGG Compound database (Kyoto Encyclopedia of Genes and Genomes, http://www.kegg.jp/kegg/compound/) and then mapped to the KEGG Pathway database (http://www.kegg.jp/kegg/pathway.html). The metabolic pathway was further confirmed by MSEA (Metabolite Set Enrichment Analysis).

### Transcriptomics

Fruit samples of RT, RM, YT and YM were obtained, and three independent biological replicates were performed. RNA was extracted from all samples using an E.Z.N.A.® Plant RNA Kit (Omega, Norcross, GA, USA), and then the quality and purity of total RNA were evaluated via an Agilent Bioanalyzer 2100 system. Wuhan Metware (Biotechnology Co., Ltd., Wuhan, China) completed the construction of the cDNA library and sequenced all the samples using an Illumina HiSeq™ 4000 system (San Diego, CA, USA).

### Functional annotation and differential genes analysis

Functional annotation was mainly based on the following databases: KEGG (Kyoto Encyclopedia of Genes and Genomes), NR (NCBI non-redundant protein sequences), SwissProt, Trembl, KOG (euKaryotic Ortholog Groups), GO (Gene Ontology), and Pfam (Protein family). Differentially expressed genes were screened by DESeq2 software. Gene expression with significant differences was screened based on the rule of false discovery rate (FDR) < 0.05 and | Log_2_FC |> 1. Furthermore, structural genes and transcription factors associated with fruit color regulation were screened from genes with significant differential expression.

### Weighted gene correlation network analysis

The hub genes were analyzed based on time-series samples to draw a differentially expressed gene (DEG) regulatory network. Standard-method-based WGCNA was performed to detect specific co-expression gene modules related to each physiological trait. In total, 41,202 differentially expressed genes were used as input expression data in this study. According to the gene expression of different sampling times, the gene expression matrix was obtained, in which the average value was used to calculate repeated sampling. A co-expression network was constructed and divided into related modules by R software (v3.4.4) and the WGCNA package (v1.6.6). The candidate genes from each module were determined according to the genetic connectivity in each module. The resulting data and data set were visualized by Cytoscape 3.5.1 and a circular layout to ensure interconnection.

### qRT‒PCR analysis

To validate the results obtained from high-throughput data, 13 color-related genes were selected for qRT‒PCR testing, and the specific details were described in our previous study [3, 40]. The specific primers for qRT‒PCR were designed using Premier V5.0, and the details of the sequences were shown in Supplementary Table 11.

### UFGT activity assay

UFGT enzyme activities were measured using reagent kits (Suzhou Keming Biotechnology Co., Ltd., China). UFGT catalyzes the formation of UDP from UDPG and quercetin; UDP oxidizes NADH to NAD^+^ under the action of pyruvate kinase and lactate dehydrogenase, the rate of NAD^+^ production is proportional to the UDP content, and the UFGT activity is reflected by the rate of decrease in absorbance at 340 nm. The crude extracts of fruits were extracted, and the UFGT enzyme activity assay was performed following the manufacturer’s instructions. Finally, the enzyme activity values were calculated. Three replicates of each sample were performed.

### Statistical analysis

All the data used in the present study were obtained from at least three biological and three technique replicates. SPSS software 16 (IBM Corporation, Armonk, NY, USA) was used for statistical analysis. One-way analysis of variance (ANOVA) was performed to determine significant differences between samples. Unless otherwise stated, data labeled with different letters represent significant differences at *P* values < 0.05. Three independent experiments were used to calculate the mean and standard error (SE).

## Supplementary Information

**Additional file 1: Figure** **S1.** The two principal components of YT VS RT (A) and YM VS RM B. **Figure S2**. Total ion flow diagram of QC samples and the multi-peak graphs of MRM metabolites. A Total ion flow diagram of QC samples (Positive ions). B Total ion flow diagram of QC samples (Negative ions). C The multi-peak graphs of MRM metabolites (Positive ions). D The multi-peakgraphs of MRM metabolites (Negative ions). **Figure S3.** Annotate all of the assembled genes in accordance with the public databases GO, Swiss-Prot, NR, COG/KOG, Trembl and KEGG. **Figure S4. **Unigenes were classified in the KOG database. **Figure S5. **The statistics of the number of differentially expressed genes and cluster analysis of transcription factors. A Statistics of the number of upregulated and downregulated DEG in the four groups of samples. The Abscissa represents the comparison between samples (groups), and the ordinate indicates the significant differentially expressed genes detected. B-C The sunburst chart of transcription factors associated with anthocyanin biosynthesis. The quantitative distribution of differentially expressed TFs in YM VS RM (B) and YT VS RT C. **Figure S6.** Top 20 of differential genes for KEGG enrichment. The ordinate is Pathway. The horizontal axis is the enrichment factor (the number of differences in this Pathway divided by all Numbers). The size is the quantity, the redder the color, the smaller the P/Q value.

**Additional file 2: Supplementary Table 1.** The raw data for the detected metabolic species in our manuscript. **Supplementary Table 2.** Differential metabolic species among the JC and NT. **Supplementary Table 3.** Summary of transcriptome sequencing data transcriptome assembly. **Supplementary Table 4.** Details of all the gene annotation. **Supplementary Table 5.** KOG function annotation of all unigenes. **Supplementary Table 6.** KEGG pathway annotation of all unigenes. **Supplementary Table 7.** Details of all the different expression genes in JC and NT.  **Supplementary Table 8.** KEGG pathways in which differentially expressed genes are involved. **Supplementary Table 9. **All DEGs of anthocyanin biosynthesis pathway. The different expression coding genes of key enzyme anthocyanin biosynthesis pathway after WGCNA. **Supplementary Table 10.** Statistical table of TFs in JC and NT. **Supplementary Table 11.** Primers used in qRT-PCR in this study.

## Data Availability

The raw RNA sequencing data obtained in this study have been uploaded to the Sequence Read Archive (SRA) in NCBI (National Center for Biotechnology Information) under the accession number PRJNA804343, https://www.ncbi.nlm.nih.gov/bioproject/PRJNA804343 and are available upon reasonable request by contacting the corresponding author (yangxiuyan@caf.ac.cn).
