# Supplementary material for: Integrated metabolome and transcriptome analysis unveils novel pathway involved in the fruit coloration of Nitraria tangutorum Bobr
Source: BMC Plant Biol. 2023 Feb 1;23:65. doi: 10.1186/s12870-023-04076-3 (PMC9890838; doi:10.1186/s12870-023-04076-3)
Supplement: Supplementary file 1 — Additional file 1: Figure S1. The two principal components of YT VS RT (A) and YM VS RM B. Figure S2. Total ion flow diagram of QC samples and the multi-peak graphs of MRM metabolites. A Total ion flow diagram of QC samples (Positive ions). B Total ion flow diagram of QC samples (Negative ions). C The multi-peak graphs of MRM metabolites (Positive ions). D The multi-peakgraphs of MRM metabolites (Negative ions). Figure S3. Annotate all of the assembled genes in accordance with the public databases GO, Swiss-Prot, NR, COG/KOG, Trembl and KEGG. Figure S4. Unigenes were classified in the KOG database. Figure S5. The statistics of the number of differentially expressed genes and cluster analysis of transcription factors. A Statistics of the number of upregulated and downregulated DEG in the four groups of samples. The Abscissa represents the comparison between samples (groups), and the ordinate indicates the significant differentially expressed genes detected. B-C The sunburst chart of transcription factors associated with anthocyanin biosynthesis. The quantitative distribution of differentially expressed TFs in YM VS RM (B) and YT VS RT C. Figure S6. Top 20 of differential genes for KEGG enrichment. The ordinate is Pathway. The horizontal axis is the enrichment factor (the number of differences in this Pathway divided by all Numbers). The size is the quantity, the redder the color, the smaller the P/Q value. [file 12870_2023_4076_MOESM1_ESM.docx]

Supplementary Material


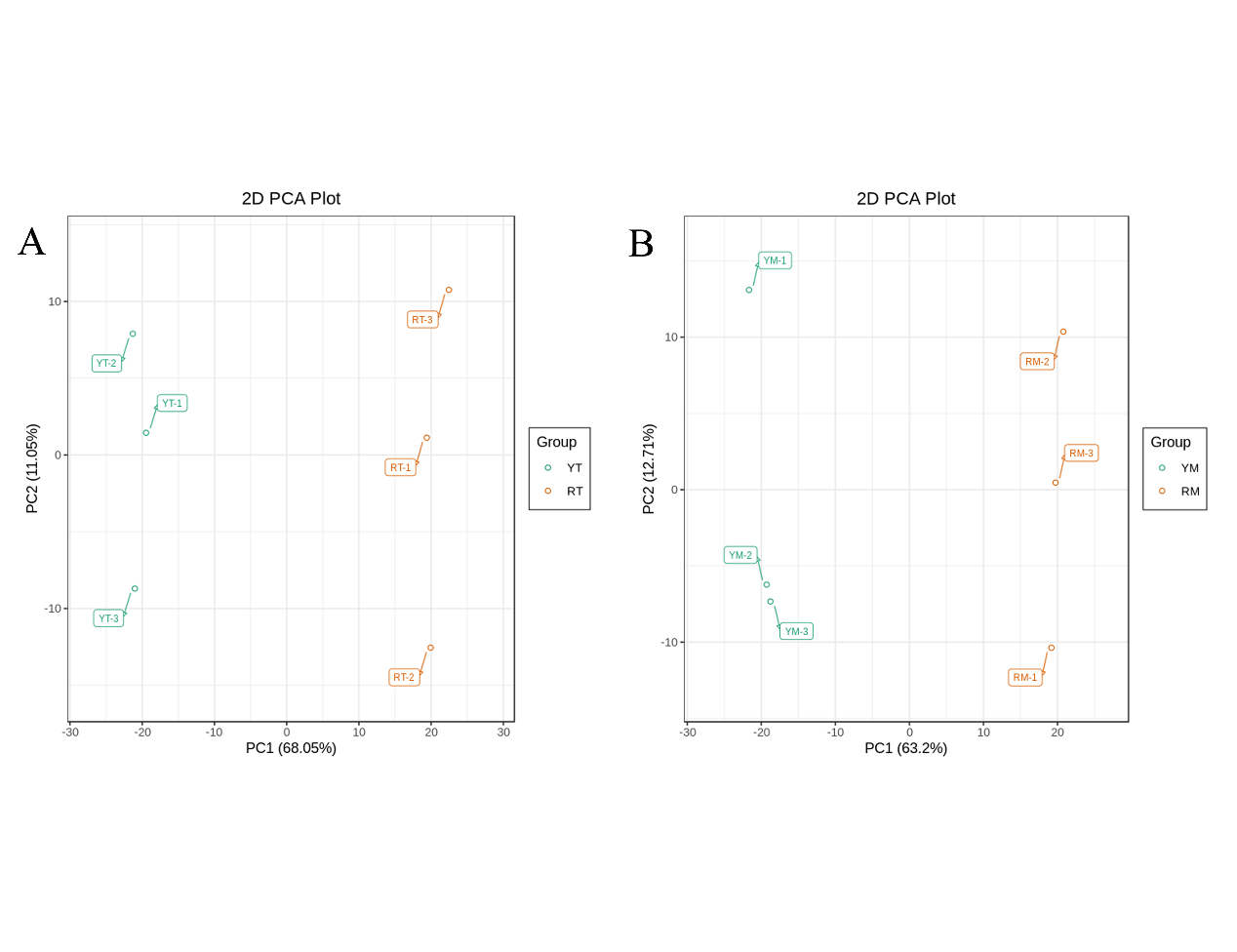


**Figure** S**1.** The two principal components of YT VS RT (A) and YM VS RM (B)


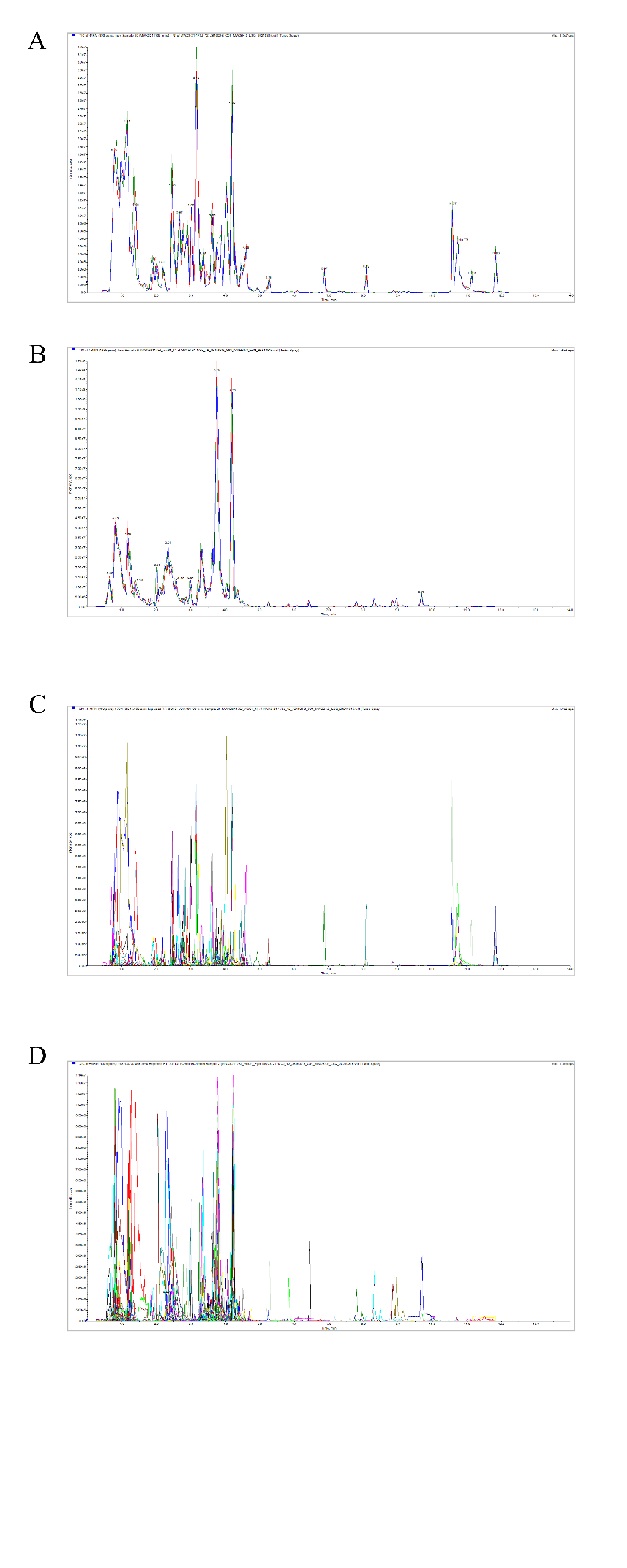


**Figure S2**. Total ion flow diagram of QC samples and the multi-peak graphs of MRM metabolites. (A) Total ion flow diagram of QC samples (Positive ions). (B) Total ion flow diagram of QC samples (Negative ions). (C) The multi-peak graphs of MRM metabolites (Positive ions). (D) The multi-peak graphs of MRM metabolites (Negative ions).


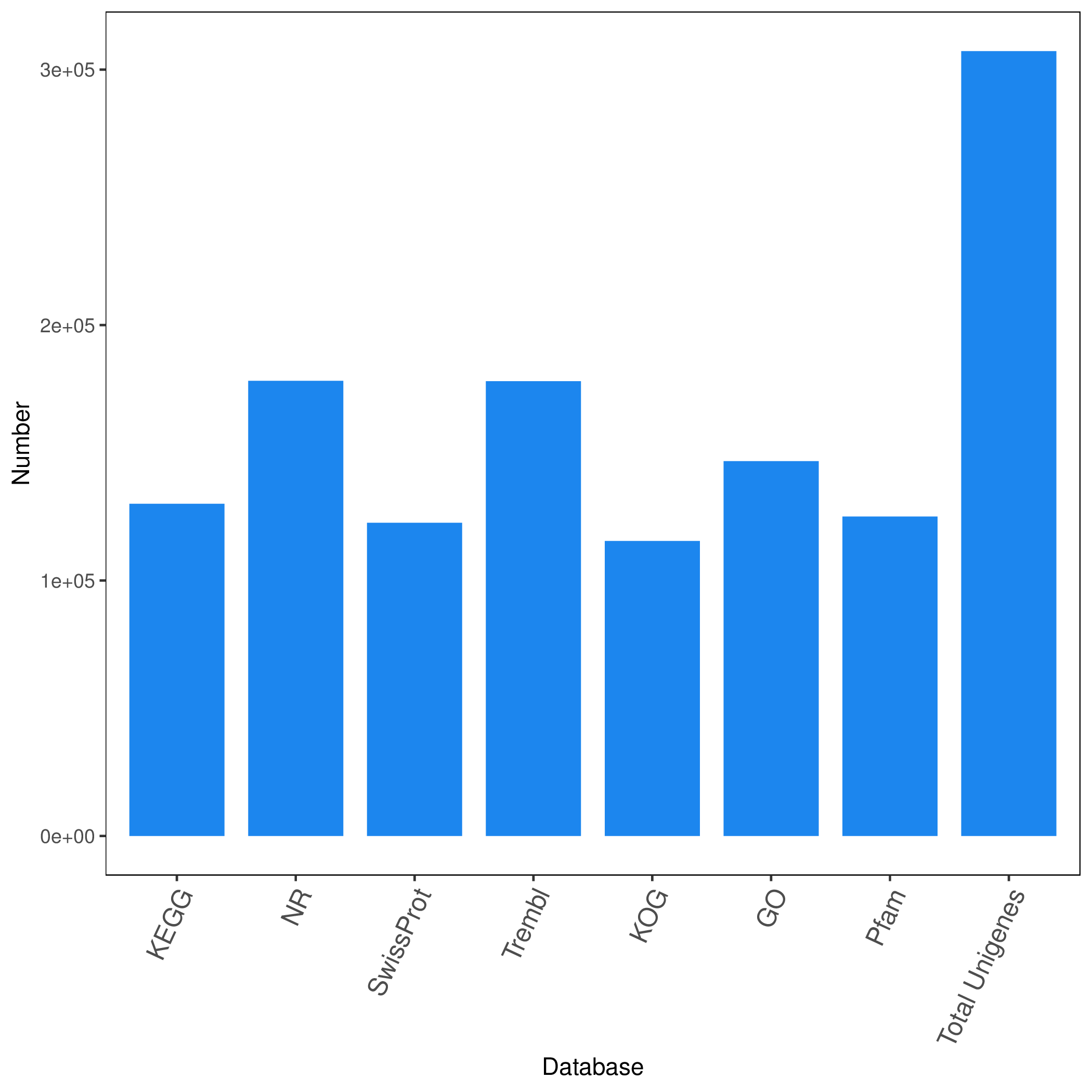


**Figure S3.** Annotate all of the assembled genes in accordance with the public databases GO, Swiss-Prot, NR, COG/KOG, Trembl and KEGG.


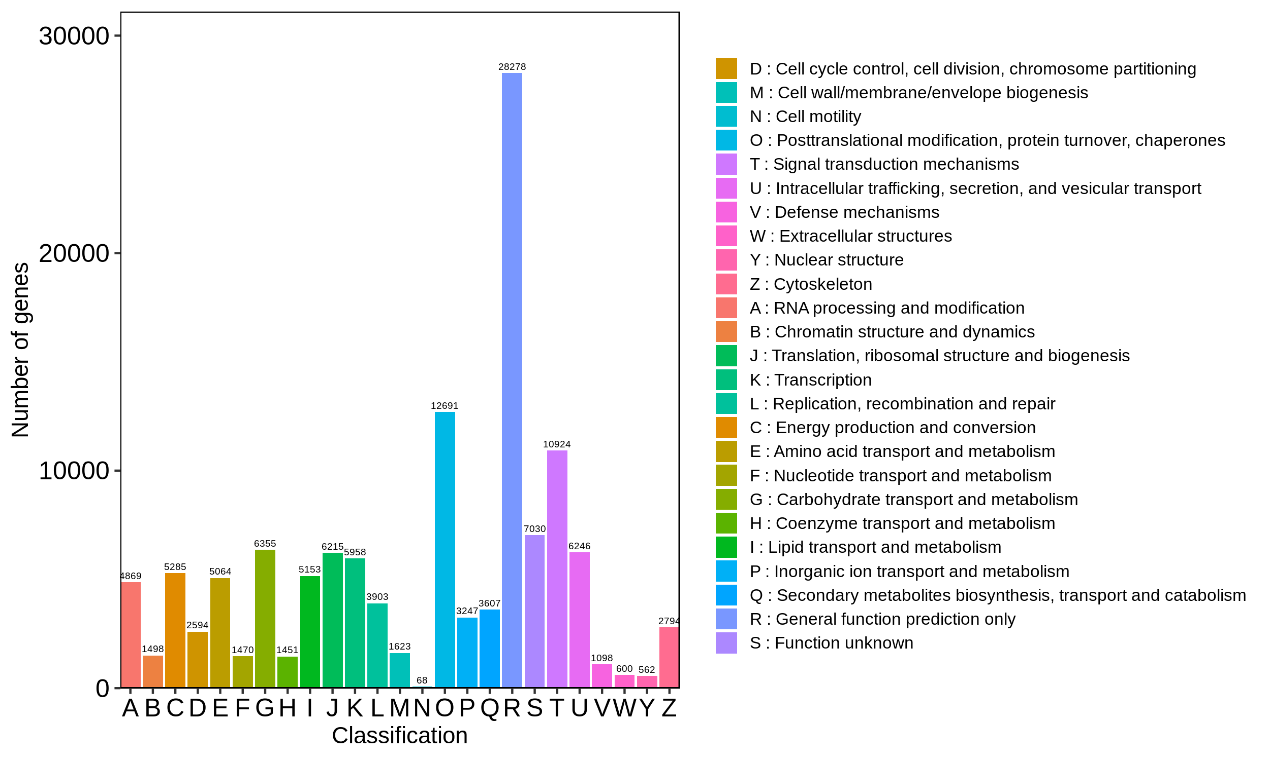


**Figure S4.** Unigenes were classified in the KOG database


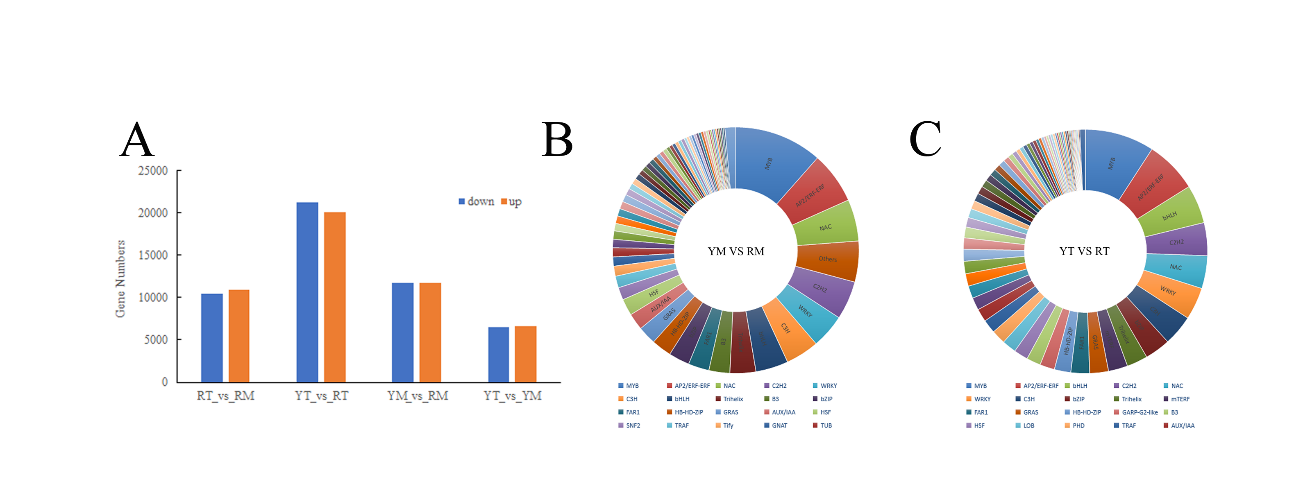


**Figure S5.** The statistics of the number of differentially expressed genes and cluster analysis of transcription factors. (A) Statistics of the number of upregulated and downregulated DEG in the four groups of samples. The Abscissa represents the comparison between samples (groups), and the ordinate indicates the significant differentially expressed genes detected. (B-C) The sunburst chart of transcription factors associated with anthocyanin biosynthesis. The quantitative distribution of differentially expressed TFs in YM VS RM (B) and YT VS RT (C).


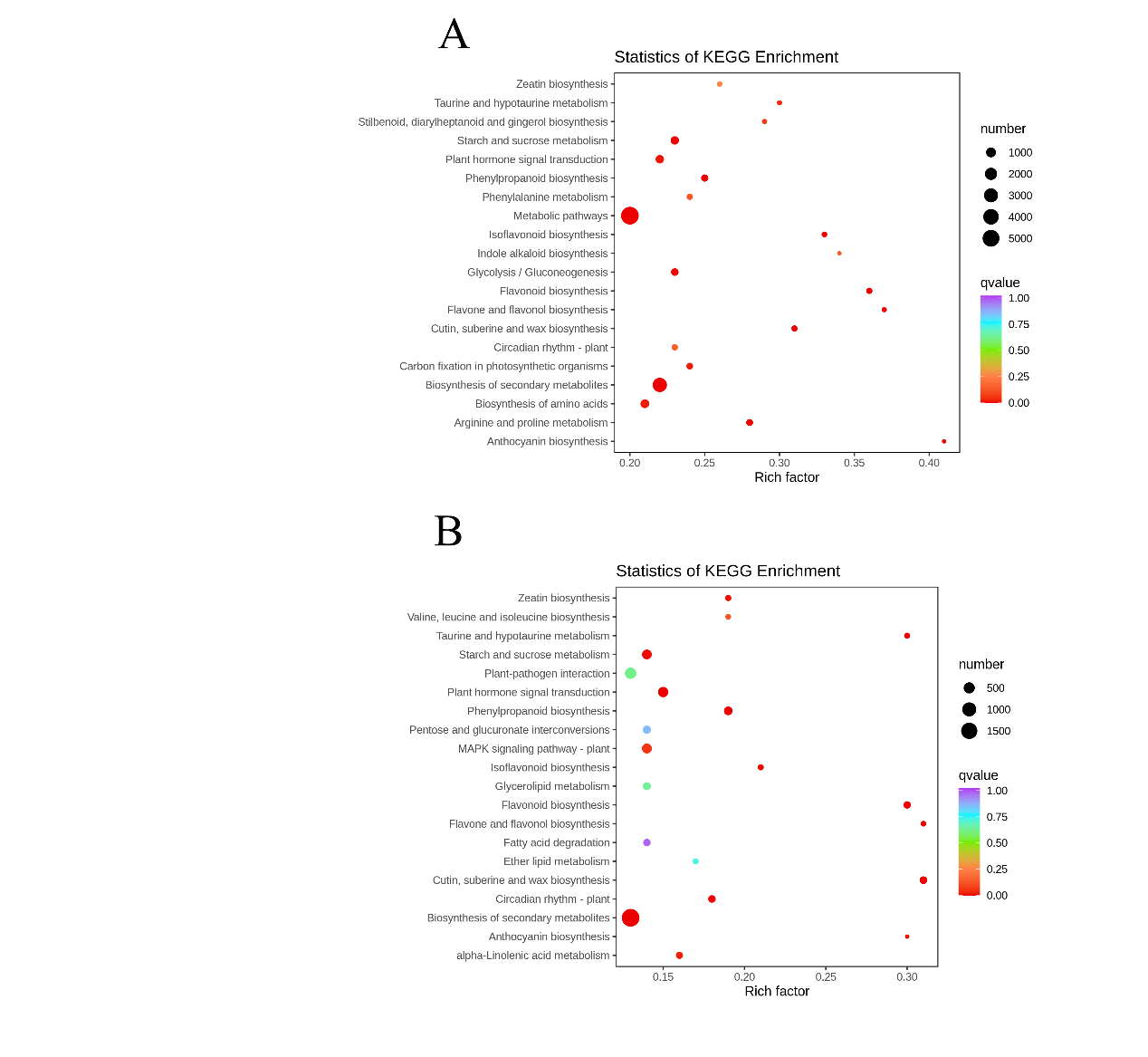


**Figure S6.** Top 20 of differential genes for KEGG enrichment. The ordinate is Pathway. The horizontal axis is the enrichment factor (the number of differences in this Pathway divided by all Numbers). The size is the quantity, the redder the color, the smaller the P/Q value.
